# Construction of an efficient delivery system for raspberry anthocyanins: preparation and stability evaluation of whey protein isolate-gum Arabic nanoparticles

**DOI:** 10.1016/j.fochx.2026.104216

**Published:** 2026-07-15

**Authors:** Chang Tan, Xue Zhang, Hanchi Ma, Chenyang Shi, Tiejing Li, Youlin Xue, Xue Peng, Xiaoqian Hu, Yanwen Kong, Chongting Guo, Mingyue Wang

**Affiliations:** a Liaoning Provincial Key Laboratory of Food and Biological Processing, Light Industry College, Liaoning University, Shenyang, Liaoning 110036, China; b College of Grain Science and Technology, Shenyang Normal University, Shenyang 110034, China; c Shenyang Medical College, School of Public Health, Liaoning Medical Functional Food Professional Technology Innovation Center, 110034, China

**Keywords:** Raspberry anthocyanins, WPI-GA composite wall materials, Stability, In vitro antioxidant activity, Protein–polysaccharide interactions, Nanoparticles

## Abstract

Raspberry anthocyanins (ACNs) show strong antioxidant and anti-inflammatory activities, but their practical application is restricted by the poor stability and low bioaccessibility. In this work, whey protein isolate and gum arabic were employed as composite wall materials to fabricate ACN-loaded nanoparticles. The nanoparticles presented a core–shell structure with high encapsulation efficiency, and their formation was driven by electrostatic and hydrophobic interactions. The nanoparticles exhibited significantly enhanced stability under different pH, ionic strength and light conditions, with an ACN retention rate above 82% after 30 days of storage. They also maintained higher ACN retention and favorable antioxidant activity during simulated gastrointestinal digestion. This study provides a feasible delivery system for the stabilization and application of raspberry anthocyanins.

## Highlights

• Raspberry ACNs were loaded into WPI–GA nanoparticles via non-covalent interactions.
• GA optimized nanoparticle morphology and enhanced stability of the delivery system.
• GA-WPI-ACN@NP showed excellent resistance to pH, ionic strength, light and storage.
• The nanosystem reduced ACN degradation and enhanced bioaccessibility in digestion.
• Encapsulated ACNs preserved high antioxidant activity.

## 1 Introduction

Raspberry (*Rubus idaeus* L., RI) is rich in anthocyanins (ACNs), primarily cyanidin-3-glucoside, cyanidin-3-rutinoside, and structurally related polyphenolic monomers (e.g., phenolic acids, flavonols). These compounds exhibit physiological functions including antioxidant and anti-inflammatory effects, and thus have broad potential in the food and pharmaceutical industries (Cui, Zhao, et al., 2025; Yuan et al., 2023). ACNs feature a typical C6–C3–C6 flavonoid skeleton, and their physiological functions are strongly dependent on the structure–activity relationship (SAR). The number and position of phenolic hydroxyl groups, glycosylation modification, and aromatic ring conjugation determine the antioxidant capacity, stability, and transmembrane transport efficiency of ACNs. Nevertheless, their practical application is severely limited by intrinsic shortcomings. First, phenolic hydroxyl groups within the ACN structure are highly susceptible to external environmental factors; exposure to light, elevated temperatures, or acidic/alkaline conditions can readily trigger degradation, oxidation, or polymerization reactions, leading to loss of bioactivity and color changes (Cahyana et al., 2022; Enaru et al., 2021; Xue et al., 2024). Second, ACNs exhibit strong polarity, with low solubility in the gastrointestinal environment, vulnerability to enzymatic degradation, and difficulty in crossing biological membrane barriers, resulting in relatively low bioaccessibility and bioavailability (Fang et al., 2020; Lila et al., 2016). Collectively, these factors restrict full exploitation of their functional value.

To overcome the poor stability and limited bioaccessibility of raspberry ACNs, various encapsulation and delivery approaches have been developed, with nanoparticle encapsulation technology attracting particular interest, owing to its unique advantages. Nanoparticle carriers can encapsulate ACNs via physical or chemical interactions, forming micro−/nanoscale core-shell particles. Such structures provide a protective barrier against environmental degradation while improving apparent solubility, modulating digestion, and facilitating absorption and utilization of active ingredients in target organs (Bordim et al., 2025; Tong et al., 2020). Among the numerous nanocarrier materials available, natural biological macromolecules have been widely used in the encapsulation and delivery of food active ingredients because of their excellent biocompatibility, high degradability, and favorable safety profile. The chain structure, cross-linking properties, and mechanical strength of natural biomacromolecules are the core factors for constructing stable delivery systems (Liu, Hou, et al., 2026; Liu, Wang, et al., 2026). Protein-polysaccharide composite systems can enhance the toughness and deformation resistance of membrane structures via molecular entanglement and cross-linking interactions. This characteristic aligns with the design philosophy for the mechanical modification of starch-based polymeric materials (Chen et al., 2025).

Whey protein isolate (WPI) is a high-quality animal-derived protein rich in *α*-lactalbumin and *β*-lactoglobulin. It offers good biocompatibility, emulsifying properties and gel-forming ability, and it is gradually degraded in the gastrointestinal environment, which can enhance the bioaccessibility of encapsulated compounds (Yadollahi et al., 2023; Yang et al., 2024). Gum arabic (GA) is a natural polysaccharide extracted from plants such as Acacia trees and is characterized by high water solubility, emulsifying properties and film-forming ability. The hydroxyl and carboxyl groups in GA can form hydrogen bonds or hydrophobic interactions with active ingredients, thereby providing a stabilizing encapsulation environment (Ansari et al., 2022; Cui, Chen, et al., 2025; Wu et al., 2018). Whey protein isolate (WPI) is an amphoteric polyelectrolyte, while gum arabic (GA) is an anionic polysaccharide. Under specific pH conditions, the WPI and GA can spontaneously form composite aggregates through electrostatic interactions, hydrogen bonds, and hydrophobic forces. The external environment and intermolecular interaction patterns directly affect the assembly behavior of the complexes (Liu, Hou, et al., 2026; Liu, Wang, et al., 2026). Based on the above properties, this study combined GA and WPI to fabricate composite wall materials for synergistically improving the structural stability of nanoparticles. The combination of WPI and GA as nanocarriers may leverage their synergistic function, balancing carrier stability and biocompatibility, and providing a new strategy for efficient encapsulation and delivery of raspberry ACNs. For example, Shaddel et al. (Shaddel et al., 2018) employed gelatin and GA as wall materials to encapsulate black raspberry ACNs via double emulsification–complex coacervation; the resulting microcapsules exhibited regular morphology and high loading capacity, and significantly reduced hygroscopicity and solubility while enhancing ACN thermal and storage stability. Mansour et al. (Mansour et al., 2020) used soybean protein isolate (SPI) and GA to microencapsulate red raspberry ACNs via an ultrasound-assisted freeze-drying method. The optimized formulation enabled more sustained release under simulated gastrointestinal conditions, effectively improving ACN bioavailability.

Despite advances in the research on raspberry ACN nanoparticles, further in-depth investigation is required to optimize the design of GA-WPI composite carriers and to elucidate the mechanisms underlying particle formation and stability regulation. Accordingly, in this study, nanoparticles were fabricated using raspberry ACNs as the core material and WPI and GA as composite wall materials. Their physicochemical properties—including particle size distribution, zeta potential, micromorphology, encapsulation efficiency, loading capacity, and molecular structure—were characterized. Furthermore, the environmental, storage, and digestive stability of the nanoparticles under various conditions were evaluated, and their in vitro antioxidant activity was assessed. This study aims to provide a theoretical basis for the stabilized application of raspberry ACNs and the development of high-value-added products.

## 2 Materials and methods

### 2.1 Materials and reagents

Raspberry samples were obtained from the Raspberry Experimental Base of Shenyang Agricultural University. GA was purchased from Yuanye Bio-Technology Co., Ltd. (Shanghai, China), and WPI (purity: 80%) was obtained from Beijing Aotoda Technology Co., Ltd. The remaining fraction mainly consists of lactose and minerals, which are considered inert components in protein-based nanoparticle assembly, in related studies. No obvious interference on nanoparticle formation was observed in the present work.

### 2.2 Extraction, purification and identification of raspberry ACNs

Raspberry ACNs were extracted according to W. Wang et al. (Wang et al., 2016). Frozen raspberry fruits were thawed under ultrasonication and homogenized using a juicer. The homogenized pulp (58 g) was mixed with 580 mL of 70% (*v*/v) ethanol at a solid-to-liquid ratio of 1:10 (g:mL), and the pH was adjusted to 3.0 with hydrochloric acid. The mixture was ultrasonicated for 2 h at 45 °C (400 W) and then stored in the dark at 4 °C for 24 h. The extract was collected by filtration, and ethanol was removed by rotary evaporation under reduced pressure to obtain a crude raspberry ACN extract. This study adopted the industry-standard ultrasonic extraction process, with reference to the process optimization methods for the ultrasound-assisted extraction of plant polyphenols (Duan et al., 2026). Preliminary experiments confirmed that the extraction yield of raspberry ACNs reached the maximum value under the conditions of 45 °C, 400 W, and 2 h. Moreover, under these conditions, the structural degradation of ACN molecules caused by high temperature and prolonged ultrasonic treatment was mitigated, thus balancing the extraction efficiency and integrity of active components.

Purification of raspberry ACNs was performed according to Bhosale et al. (Bhosale et al., 2025). A pretreated AB-8 resin was packed into a glass column (Φ16 × 400 mm) with an aspect ratio of 15:1 and operated at a flow rate of 1.0 BV/h. A 10-mL aliquot of the crude ACN extract was diluted to 100 mL with citric acid–sodium citrate buffer (pH 2.5) and loaded onto the column. Elution was performed using 60% ethanol at pH 1.5. Fractions were collected according to the colored band, while elution was monitored at 520 nm. To ensure complete recovery of residual ACNs, a second elution was performed with 150 mL of the same eluent. The eluates were combined and concentrated by rotary evaporation under reduced pressure at 40 °C, followed by freeze-drying to obtain purified raspberry ACNs, which were stored at 4 °C until further use.

Component identification of the purified raspberry ACNs was conducted by liquid chromatography-tandem mass spectrometry (LC-MS/MS) using a Waters H-Class ultra-performance liquid chromatography (UPLC) system (Waters, USA) coupled to a 5600 Q-TOF high-resolution mass spectrometer (HRMS) equipped with an ESI ion source (AB SCIEX, USA). Separation was performed on a Waters ACQUITY UPLC HSS T3 column (1.8 μm, 2.1 × 100 mm; Waters, USA). Acetonitrile, methanol, formic acid, and other reagents were of analytical grade. Sample preparation involved dissolution of the lyophilized sample (3.6 mg) in 75% methanol aqueous solution. Freeze-drying may lead to the formation of microaggregates of the ACN particles. Hence, the solution was ground to reduce the particle size, ultrasonicated to dissociate microaggregates and promote complete dissolution, centrifuged (17,000 ×*g*, 20 °C) to remove trace insoluble fine particles, and filtered through a 0.22-μm membrane before injection in order to avoid chromatographic column blockage and mass-spectrometer ion-source contamination. The samples were analyzed under the chromatographic and mass spectrometric conditions described above at a flow rate of 400 μL/min. Raw data were converted and processed, and compound identification was performed by matching against multiple databases using mass-to-charge ratio (*m*/*z*) error and preset identification thresholds.

### 2.3 Nanoparticle preparation

#### 2.3.1 Whey protein isolate nanoparticles (WPI@NP)

WPI nanoparticles were prepared according to Salah et al. (Salah et al., 2020) with minor modifications. Briefly, WPI (1 g) was dissolved in 100 mL of 10 mmol/L sodium chloride, and the pH was adjusted to 7.0 with 1 mol/L sodium hydroxide. The solution was magnetically stirred at 1126 rpm for 2 h and incubated at 4 °C for 12 h under ambient humid conditions. The relative humidity was neither monitored nor controlled during the incubation step. The mixture was then filtered through a 0.45-μm membrane to remove protein aggregates. Ethanol was added dropwise to the filtrate at 1 mL/min. The turbidity endpoint was determined via standardized visual inspection under consistent laboratory ambient light and fixed background conditions. The endpoint was defined as the first occurrence of persistent homogeneous visible cloudiness in the solution, indicating the onset of protein colloidal assembly. Next, 0.5 mL of 1-ethyl-3-(3-dimethylaminopropyl) carbodiimide (EDC, 6 mg/mL) solution was added, and the reaction was allowed to proceed for 3 h under constant stirring. EDC was employed as a zero-length crosslinking agent to activate the carboxyl groups of WPI and mediate covalent crosslinking between the amine and carboxyl residues of the whey protein molecules, thereby reinforcing the structural integrity and colloidal stability of the formed WPI nanoparticles against environmental variations. The reaction mixture was centrifuged at 10000*g* for 25 min, and the precipitate was collected and resuspended in 10 mmol/L citric acid–sodium citrate buffer (pH 3.0), followed by dilution to a final volume of 100 mL. The suspension was sonicated for 7 min in an ice-water bath at 260 W and stored on ice after cooling. The residual unreacted EDC was eliminated via sequential high-speed centrifugation, dialysis, and freeze-drying treatments. The final nanoparticle products contained no detectable EDC residue, were non-toxic, and met food-grade safety requirements.

#### 2.3.2 Whey protein isolate–anthocyanin nanoparticles (WPI-ACN@NP)

A 10-mL aliquot of raspberry ACN solution (3.88 mg/mL) was added dropwise to 10 mL of WPI@NP suspension (pH 3.0) at 1 mL/min, and the mixture was magnetically stirred at 1126 rpm for 1 h. In this study, ACN was loaded onto the surface of preformed WPI nanoparticles through electrostatic interactions and hydrogen bonding rather than co-assembly. The ACN concentration (3.88 mg/mL) was determined based on preliminary gradient screening tests. This optimal concentration led to high ACN loading efficiency while preventing particle aggregation and precipitation caused by excessive ACN dosage, thus balancing loading capacity and colloidal stability. The resulting suspension was centrifuged at 10000 ×*g* for 20 min. The precipitate was resuspended in 10 mmol/L citric acid–sodium citrate buffer (pH 3.0) and sonicated for 7 min in an ice-water bath at 260 W. After cooling, the sample was stored in an ice bath until further analysis.

#### 2.3.3 Gum Arabic–whey protein isolate–anthocyanin nanoparticles (GA-WPI-ACN@NP)

GA was dissolved in 10 mmol/L citric acid–sodium citrate buffer (10 mL, pH 3.0) to obtain a GA solution with a concentration of 15 mg/mL. The GA concentration was selected according to pre-experiment optimization to form a dense and uniform polysaccharide coating layer, without causing excessive viscosity or particle flocculation. Next, 10 mL of WPI-ACN@NP suspension was added dropwise to the GA solution at 1 mL/min, followed by magnetically stirring at 1126 rpm for 1 h. In this ternary system, GA further coated the outer layer of the WPI-ACN nanoparticles via electrostatic adsorption, constructing a core-shell structured ternary nanocarrier. After the reaction, the mixture was centrifuged at 10000*g* for 20 min. The precipitate was resuspended in the same citric acid–sodium citrate buffer, sonicated in an ice-water bath at 260 W for 7 min, and stored in an ice bath after cooling.

### 2.4 Determination of ACN content

ACN content was determined using the pH differential method (Taghavi et al., 2022). Samples were diluted separately with 0.025 M potassium chloride buffer (pH 1.0) and 0.4 M sodium acetate buffer (pH 4.5). Absorbance was measured at 520 and 700 nm, using sodium acetate buffer as the blank reference. The total ACN content (C) at pH 1.0 and pH 4.5 was calculated according to the following equations:

(1) $$A={\left(A_{520\mathrm{nm}}-A_{700\;\mathrm{nm}}\right)}_{\mathrm{pH}1.0}-{\left(A_{520\mathrm{nm}}-A_{700\;\mathrm{nm}}\right)}_{\mathrm{pH}4.5}$$

(2) $$C\left(\mathrm{mg}/\mathrm{mL}\right)=\frac{A\times M_{W}\times \mathrm{DF}}{\varepsilon \times L}$$

where *M*_*w*_ = 449.2 g/mol and *ε* = 26,900 are the cyanidin-3-O-glucoside relative molecular weight and molar extinction coefficient, respectively; *DF* is the sample dilution factor; and *L* is the cuvette path length.

### 2.5 Determination of ACN encapsulation efficiency (EE) and loading capacity (LC)

The encapsulation efficiency (EE) and loading capacity (LC) of the ACN nanocomposites were determined by ultrafiltration centrifugation, according to a previously published protocol (Jiang et al., 2022). An appropriate amount of ACN nanocomposites was loaded into the inner tube of an Amicon Ultra-15 ultrafiltration centrifugal tube (MilliporeSigma, St. Louis, MI, USA) and centrifuged at 4000 ×*g* and 4 °C for 40 min. After centrifugation, the ACNs detected in the filtrate were considered free (unencapsulated) ACNs. The retained retentate containing ACN-loaded nanocomposites was collected and lyophilized to constant weight for obtaining the total dry mass of the nanocomposites.

EE (%) and LC (%) were calculated according to Eqs. (3), (4), respectively.

(3) $$\mathrm{EE}\left(\%\right)=\frac{\mathrm{TotalACNs}-\mathrm{FreeACNs}}{\mathrm{TotalACNs}}\times 100\%$$

(4) $$\mathrm{LC}\left(\%\right)=\frac{\text{Total ACNs}-\text{Free ACNs}}{\mathrm{Dry}\;\text{mass of}\;\mathrm{ACN}\;\text{nanocomposites}}\times 100\%$$

where *Total ACNs* is the ACN content initially added to prepare the ACN nanoparticles (mg/mL), *Free ACNs* is the ACN content in the filtrate (mg/mL), and *Dry mass of ACN nanocomposites* is the total dry weight of the lyophilized ACN-loaded nanocomposites (mg).

### 2.6 Structural characterization of nanoparticles

#### 2.6.1 Dynamic light scattering (DLS) analysis

The mean particle size, polydispersity index (PDI), and zeta potential of the nanoparticles were determined at 25 °C using a NanoZS90 particle size analyzer (Malvern Panalytical, UK). Samples were diluted to an appropriate concentration with 10 mmol/L citric acid–sodium citrate buffer (pH 3.0) before analysis.

#### 2.6.2 Fluorescence spectroscopy analysis

Fluorescence spectra were recorded at 298 K using an F-7000 fluorescence spectrophotometer (Hitachi, Japan). The excitation wavelength was set to 280 nm, and emission spectra were collected from 300 to 450 nm with a 5-nm slit width and a scanning speed of 1200 nm/min. Citric acid–sodium citrate buffer (pH 3.0) was used to zero the instrument.

#### 2.6.3 Ultraviolet–visible spectroscopy (UV–vis) analysis

UV–Vis absorption spectra were obtained at 298 K using a UV-1600 spectrophotometer (Mapada Instruments Co., Ltd., China) over the range of 190–450 nm.

#### 2.6.4 Fourier transform infrared spectroscopy (FTIR) analysis

Freeze-dried nanoparticle samples (5 mg) were ground with KBr at a 1:100 (*w*/w) ratio and pressed into pellets. Fourier transform infrared (FTIR) spectra were collected using a TENSOR II FTIR spectrometer (Bruker Corporation, Germany), with air as the background. Measurements were performed over 4000–400 cm^−1^ at a resolution of 2 cm^−1^.

#### 2.6.5 Circular dichroism spectroscopy (CD) analysis

Circular dichroism (CD) spectra were acquired using a J-810 CD spectrometer (JASCO Corporation, Japan), with 10 mmol/L citric acid–sodium citrate buffer (pH 3.0) as the blank. Measurements were conducted in a 1-mm cuvette over 190–300 nm at a scanning speed of 100 nm/min and a data interval of 1 nm.

#### 2.6.6 X-ray diffraction (XRD) analysis

Samples were equilibrated at room temperature at 28.8% relative humidity for 24 h prior to analysis. X-ray Diffraction (XRD) measurements were performed using an Xpert X-ray diffractometer (PANalytical B.V., the Netherlands) with a monochromatic Cu-K*α* radiation source (λ = 0.1542 nm) operated at 40 kV and 40 mA. Data were acquired over 3–45° with a step size of 0.02° and a scanning rate of 4°/min.

#### 2.6.7 Transmission electron microscopy (TEM) analysis

A drop of the freshly prepared nanoparticle suspension was deposited onto a copper grid, air-dried at room temperature, and imaged using a FEI Tecnai G2 Spirit transmission electron microscope (TEM) (FEI Company, USA) operated at an accelerating voltage of 100 kV and 20,000× magnification.

#### 2.6.8 Atomic force microscopy (AFM) analysis

The samples were diluted with deionized water, deposited onto a clean mica sheet, and dried at room temperature. The surface morphology and roughness of the nanoparticles were observed in tapping mode using a Bruker Dimension Icon atomic force microscope (AFM) (Bruker Corporation, Germany) with a scan area of 5 μm × 5 μm, a scan rate of 1 Hz, and an accelerating voltage of 10 kV.

#### 2.6.9 Molecular docking simulation

AutoDock Vina was used to simulate the interactions between *β*-lactoglobulin and ACN monomers (cyanidin-3-*O*-glucoside, delphinidin-3-*O*-rutinoside, pelargonidin-3-glucoside). After structural pretreatment including dehydration, hydrogen network optimization, and energy minimization, the protein and ligand files were converted into PDBQT format. The exhaustiveness was set to 32, and ten conformations were generated for each group. The optimal conformation with the lowest binding free energy was chosen to evaluate the binding affinity and binding sites.

### 2.7 Stability analysis of nanoparticles

#### 2.7.1 Environmental stability

Nanoparticle stability under different environmental conditions (pH, ionic strength, and light exposure) was evaluated according to the method reported by Tao et al. (Tao et al., 2025). Freshly prepared nanoparticle dispersions were adjusted to different pH values (2.0–8.0) using 1 M HCl or 1 M NaOH, followed by homogenization. After equilibration for 10 min, the average particle size, PDI, zeta potential of each sample were measured.

The ionic strength stability of the nanoparticles was evaluated in sodium chloride (NaCl) solutions of different concentrations at pH 3.0. NaCl was added to 5 mL of nanoparticle dispersion to obtain final NaCl concentrations of 0–100 mM. After equilibration at 25 °C for 12 h, the average particle size, PDI, zeta potential of the nanoparticle dispersions were determined.

For light stability testing, samples were incubated in a WH-10 constant-temperature light incubator (Wiggens, Germany) and subjected to light treatment for 7 days under controlled ambient conditions (room temperature, 5000 lx light intensity, and 50 μW ultraviolet intensity). Aliquots were collected on Days 0, 1, 3, 5, and 7, and the mean particle size, PDI, and zeta potential of each sample were measured.

#### 2.7.2 Storage stability

Storage stability of the nanoparticle dispersions was evaluated according to the method reported by L. Li et al. (Li et al., 2021) with minor modifications. Under two different storage conditions: 30 days at 4 °C or 30 days at 25 °C. The average particle size, PDI, zeta potential and of the resulting nanoparticle dispersions were determined on days 4, 7, 15, 21 and 30.

#### 2.7.3 Digestive stability

The in vitro release behavior and digestive stability of ACN nanoparticles under simulated gastric fluid (SGF) and simulated intestinal fluid (SIF) conditions were investigated according to the method reported by Correa-Betanzo et al. (Correa-Betanzo et al., 2014) with minor modifications.

The in-vitro simulated digestion was conducted in a constant-temperature shaker at 37 °C. Solutions of 6 M HCl and 0.9 M NaHCO₃ were prepared in advance for pH adjustment. For the gastric phase, 0.25 mL of SGF was added per 10 mL of sample and the pH of the system was adjusted to 3.0; samples were collected after 1 and 2 h of gastric digestion. For the intestinal phase, 2.5 mL of SIF was added per 10 mL of the gastric-digested sample and the pH was adjusted to 7.5; samples were collected after 1 and 2 h of intestinal digestion. At each time point, samples were centrifuged at 10,000 ×*g* for 10 min at 4 °C. The supernatant was collected, and the residual ACN concentration was quantified according to the method described in Section 2.4 to assess digestive stability. The ACN retention in the nanoparticles was calculated using Eq. (4).

(5) $$\mathrm{ACN}\;\mathrm{Retention Rate}\left(\%\right)=\frac{C_{t}}{C_{o}}$$

where *C*_*t*_ and *C*_*0*_ represent the ACN contents at the gastric/intestinal digestion time point and at the initial time point, respectively (mg/mL).

The remaining supernatants were stored at −80 °C for subsequent analyses.

### 2.8 In vitro antioxidant analysis of anthocyanin nanoparticles

#### 2.8.1 ABTS cation radical scavenging activity

The ABTS cation radical scavenging activity of the samples was determined with reference to the method of Cui, Zhao, et al. (2025). The ABTS stock solution was prepared by mixing 7 mM ABTS with 2.45 mM potassium persulfate at a 1:1 (*v*/v) ratio and allowing the mixture to stand overnight at room temperature in the dark. The ABTS stock solution was then serially diluted with absolute ethanol to an absorbance of 0.70 ± 0.02 at 734 nm to obtain the ABTS working solution. Next, 20 μL of the sample solution was thoroughly mixed with 3.0 mL of the ABTS working solution and incubated at room temperature in the dark for 15 min. The absorbance of the reaction system was then measured at 734 nm, and the ABTS cation radical scavenging activity was calculated using Eq. (5).

(6) $$\mathrm{ABTS Cation Radical Scavenging Rate}\%=\frac{A_{0}-A_{1}}{A_{0}}\times 100$$

where *A*_*0*_ is the absorbance of the blank control (sample replaced with deionized water) at 734 nm and *A*_*1*_ is the absorbance of the sample at 734 nm.

#### 2.8.2 Ferric reducing power

The ferric reducing power of the samples was determined according to the method reported by Kong et al. (Kong et al., 2023) with minor modifications. The sample solution (1 mL) was thoroughly mixed with 1 mL of 1 mM PBS (pH 6.6) and 1 mL of potassium ferricyanide solution, and incubated in a constant-temperature water bath at 50 °C for 20 min. The reaction was terminated by immediately adding 1 mL of trifluoroacetic acid solution, followed by centrifugation at 7000 ×*g* for 10 min. After centrifugation, 2 mL of the supernatant was collected, and 2 mL of deionized water and 400 μL of ferrous chloride solution were added sequentially. The mixture was vortexed thoroughly and allowed to stand at room temperature for 10 min. Subsequently, the absorbance of the reaction solution was measured at 700 nm, and the ferric reducing power was calculated using Eq. (6).

(7) $$\mathrm{Ferric Reducing Power}\left(\mathrm{mmol}\;\mathrm{Fe}\;\left(\mathrm{II}\right)\;\mathrm{eq}/g\right)=\frac{C\times V_{T}\times D}{m\times \mathrm{Vs}}$$

where *C* (mmol) is the Fe^2+^ concentration obtained from the FeSO₄ standard curve based on sample absorbance; *V*_*T*_ (mL) is the total volume of the reaction system in which the supernatant participates in the subsequent reaction; *D* is the sample dilution factor; *m* (g) is the mass corresponding to 1 mL of the raspberry ACN nanoparticle sample; and V_S_ (mL) is the sample volume used in the reaction.

#### 2.8.3 Hydroxyl radical scavenging activity

The hydroxyl radical scavenging activity of the samples was determined according to the method reported by L. Li et al. (Li et al., 2021) with minor modifications. Briefly, 100 μL of the sample was mixed with 1 mL of PBS buffer, 1 mL of ferrous sulfate solution, 1 mL of salicylic acid solution, and 1 mL of H₂O₂ solution, and the final volume was adjusted to 5 mL with distilled water. For the blank group, H₂O₂ was replaced with distilled water; for the control group, the sample was replaced with distilled water. The mixtures were incubated in a water bath at 37 °C for 30 min and then immediately cooled to terminate the reaction. The absorbance of the samples was measured at 510 nm, and the hydroxyl radical scavenging activity was calculated using Eq. (7).

(8) $$\mathrm{Hydroxyl Radical}\;\left(\cdot \mathrm{OH}\right)\;\mathrm{Scavenging Rate}\%=\left(1-\frac{A_{2}-A_{0}}{A_{1}}\right)\times 100$$

where *A*_*0*_, *A*_*1*_, and *A*_*2*_ are the absorbances of the blank, control, and experimental groups at 510 nm, respectively.

### 2.9 Statistical analysis

All experiments were performed in triplicate, and results are presented as the mean ± standard deviation (SD). Statistical analysis was conducted using SPSS 26.0 using one-way analysis of variance (ANOVA), with *p* < 0.05 considered statistically significant. Origin 2021 was used to plot all the figures and charts.

## 3 Results and discussion

### 3.1 Identification and analysis of raspberry ACNs

A total of 34 major compounds were detected from the purified raspberry extract via LC–MS/MS. Compounds 1–28 were clearly identified with mass ppm errors <5 ppm. Compounds 29–34 exhibited ppm errors greater than 10 ppm and were tentatively assigned due to limited fragment information. The detailed identification results are presented in Table S1. Based on the LC-MS/MS identification results of the purified sample, the target raspberry ACN species (cyanidin-3-*O*-glucoside and delphinidin-3-*O*-rutinoside) were clearly detected with high-confidence spectral matching under the adopted purification workflow. Thus, the applied extraction and purification protocol enables the acquisition of samples containing the characteristic target ACNs of raspberry. Two principal ACNs were clearly identified: cyanidin-3-O-glucoside (retention time: 7.356 min; relative peak area: 2.85%) and delphinidin-3-O-rutinoside (retention time: 11.628 min; relative peak area: 2.37%). Both are characteristic naturally occurring ACNs (Renai et al., 2021). As shown in Fig. S1, the protonated molecular ions [M + H]^+^ were observed in full-scan MS spectra at *m/z* 449.1032 and 611.1605. The diagnostic fragment ions obtained from MS/MS (tandem mass spectrometry) spectra showed high consistency with the database reference spectra (mass errors <10 ppm). Chemically, all polyphenolic components in the extract can be systematically categorized into anthocyanin and non-anthocyanin polyphenol fractions. Besides the two major ACNs identified, the other annotated compounds were predominantly flavan-3-ols, phenylpropanoids, ellagic acid, and flavonoid glycosides, which represented the other dominant detectable constituents. Detailed spectral information, retention times, and relative contents of all non-ACN components are summarized in Table S1. Notably, flavan-3-ols such as procyanidin B1 and catechin were successfully detected. These substances can synergize with ACNs to improve the antioxidant capacity and structural stability of the nanocarrier system. For antioxidant synergy, flavan-3-ols participate in redox cycling with anthocyanins: they rapidly donate hydrogen protons to regenerate oxidized anthocyanin flavylium cations, extend the free radical scavenging duration of the system, and exert superimposed antioxidant efficacy through multi-site radical capture (Quan et al., 2025). For colloidal stability, the polyhydroxyl structure of flavan-3-ols forms multiple hydrogen bonds and π-π stacking interactions with both anthocyanin molecules and nanocarrier biomacromolecules, which enhances the interfacial film strength of nanoparticles, inhibits particle aggregation, and improves the physical stability of the colloidal system during storage (Zhu et al., 2024). Phenylpropanoids, including gallic acid and caffeic acid, also exhibited strong free-radical-scavenging activity. Their ortho-diphenol structures can effectively scavenge reactive oxygen intermediates generated during anthocyanin degradation, interrupt the chain oxidation reaction, and indirectly reduce the oxidative decomposition rate of anthocyanins, thus constructing a cascade antioxidant defense system together with anthocyanins (Demirci & Jafari, 2026). Ellagic acid, a polyphenolic component, may improve ACN stability, specifically through dual protective mechanisms: it forms non-covalent co-assembly complexes with anthocyanins via hydrophobic interaction and hydrogen bonding to shield the vulnerable C4 site of the flavylium ring from nucleophilic attack by water molecules and enzymes, and scavenges surrounding oxidative free radicals to inhibit the ring-opening degradation pathway of anthocyanins, thus enhancing both chemical stability of anthocyanins and structural stability of the colloidal system(Wu et al., 2022); while flavonoid glycosides further enrich the bioactivity profile of the extract, in addition, flavonoid glycosides with moderate amphipathicity can adsorb on the nanocarrier surface to regulate interfacial charge and hydrophilicity, increase inter-particle steric hindrance, and assist in maintaining uniform dispersion of the colloidal system during storage (Davidson et al., 2023). Collectively, these components provide a compositional basis for subsequent investigations of the antioxidant, anti-inflammatory, and other bioactivities of raspberry ACN nanoparticles.

### 3.2 Particle size and zeta potential of nanoparticles

The particle size, zeta potential, and polydispersity index (PDI) of different nanoparticles were analyzed (Table 1), alongside the encapsulation efficiency (EE) and loading capacity (LC) of the ACNs. WPI self-assembled into positively charged nanoparticles (WPI@NP) with a zeta potential of 22.1 mV, consistent with the results of prior research (Salah et al., 2020). After ACN loading to form WPI-ACN@NP, the particle size increased from 292.1 to 397.9 nm (Fig. S2B, C). The PDI remained unchanged, while the zeta potential slightly increased (p < 0.05), indicating successful ACN binding to the protein matrix via hydrophobic interactions and hydrogen bonds (Cai et al., 2022). The binary WPI-ACN@NP achieved an EE of 68% and an LC of 275 mg/g solely via surface adsorption. Loosely adsorbed ACNs readily detached during centrifugation and degraded the overall encapsulation performance. Further coating with GA generated core–shell GA-WPI-ACN@NP with a larger particle size of 448 nm and reversed surface charge (−10.4 mV). The GA shell captured free ACNs through electrostatic attractions and intermolecular hydrogen bonds, thus increasing EE and LC to 81% and 302 mg/g, respectively. In accordance with the classical DLVO (Derjaguin-Landau-Verwey-Overbeek) colloidal stability theory, colloidal systems with an absolute zeta potential above 30 mV can maintain long-term stability predominantly through electrostatic repulsion, as the electrostatic energy barrier effectively overcomes interparticle van der Waals attraction (Wu et al., 2025). The ternary nanoparticles exhibited weak electrostatic repulsion due to their low charge magnitude, which alone was insufficient to support stable colloidal dispersion. However, the adsorbed long GA polysaccharide chains formed a dense hydrated layer on the particle surface, inducing strong steric repulsion to prevent particle collision, aggregation and sedimentation following the steric stabilization principle. The charge reversal also validated the successful GA coating, as the abundant carboxyl groups of GA led to a negatively charged outer surface. At the preparation pH of 3.0, all the samples displayed narrow size distributions, with the PDI confined to the range of 0.23–0.25. As summarized in Table 1, WPI-ACN@NP suffered severe aggregation at pH 4–5 near the isoelectric point of WPI, with a peak PDI of 0.666 at pH 5. This phenomenon aligns with DLVO theory: the near-neutral surface charge at the isoelectric point eliminates electrostatic repulsion, causing van der Waals attraction to dominate and induce significant particle flocculation, accompanied by notable ACN dissociation and poor retention. In contrast, GA-WPI-ACN@NP retained a low PDI and stable particle dimensions across this pH range, demonstrating that GA-derived steric hindrance is independent of pH conditions and indispensable for preventing pH-triggered flocculation (Wu et al., 2025).

**Table 1:** Average particle size, polydispersity index (PDI), and zeta potential of different nanoparticles.⁎

Nanoparticles	PH	Mean Particle Size (nm)	PDI	Zeta Potential (mV)	EE (%)	LC (mg/g)
WPI@NP	3.0	292.1 ± 3.63^c^	0.234 ± 0.02^b^	22.1 ± 0.2^b^	–	–
WPI-ACN@NP	3.0	397.9 ± 5.45^b^	0.245 ± 0.03^a^	23.7 ± 0.1^a^	68	275
GA-WPI-ACN@NP	3.0	448 ± 4.98^a^	0.243 ± 0.04^a^	−10.4 ± 0.2^c^	81	302
WPI@NP	4.0	352.6 ± 6.81^b^	0.379 ± 0.00^a^	1.8 ± 0.3^b^	–	–
WPI-ACN@NP	4.0	451.3 ± 8.22^a^	0.276 ± 0.004^b^	1.5 ± 0.2^b^	–	–
GA-WPI-ACN@NP	4.0	462.5 ± 7.63^a^	0.316 ± 0.003^a^	−8.2 ± 0.4^a^	–	–
WPI@NP	5.0	1128.5 ± 92.4^a^	0.398 ± 0.00^a^	0.8 ± 0.2^b^	–	–
WPI-ACN@NP	5.0	1562.3 ± 115.6^a^	0.666 ± 0.004^a^	0.5 ± 0.3^b^	–	–
GA-WPI-ACN@NP	5.0	455.2 ± 6.35^b^	0.136 ± 0.002^b^	−9.5 ± 0.3^a^	–	–

⁎ Different lowercase letters in the same column indicate significant differences (*p* < 0.05).

### 3.3 Fluorescence spectroscopy analysis, thermodynamic analysis and molecular docking

**Fig. 1:**
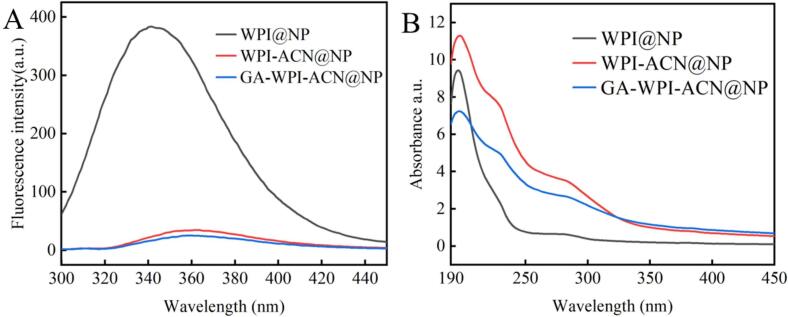
Fluorescence emission spectra (A) and UV–Vis absorption spectra (B) of different nanoparticles.

Fluorescence spectroscopy is mainly used to analyze the interactions between proteins and other components within the complexes. Proteins exhibit intrinsic fluorescence, which is primarily derived from tryptophan (Trp) and tyrosine (Tyr), with Trp exhibiting the strongest fluorescence intensity with a maximum emission wavelength at approximately 340 nm (Zang et al., 2024). Trp is the dominant fluorophore in naturally isolated whey protein. As shown in Fig. 1A, the characteristic peak of WPI was at 338 nm after self-assembly into nanoparticles, suggesting that Trp was located in a relatively nonpolar environment, consistent with the folded conformation of natural whey protein. ACN addition markedly decreased the fluorescence intensity of WPI@NP and induced a red shift from 338 to 360 nm, indicating pronounced fluorescence quenching. This effect may be attributed to the formation of nonfluorescent complexes between the ACN and Trp residues in proteins, and greater exposure of Trp residues to a more hydrophilic microenvironment (Niu et al., 2015). Subsequent GA addition further reduced nanoparticle fluorescence intensity, suggesting tighter interactions or more extensive complex formation among WPI@NP and ACN, although the hydrophobic microenvironment around the fluorophores did not appear to increase. Similarly, Wan et al. (Wan et al., 2023) reported a gradual decrease in the fluorescence intensity of rice glutelin upon GA addition and proposed static quenching during polysaccharide–protein complex formation. Therefore, the reduced fluorescence observed here may likewise result from partial shielding or encapsulation of Trp residues through protein–protein and protein–polysaccharide interactions (Luo et al., 2021).

To further clarify the binding characteristics between ACN and WPI, we calculated the corresponding thermodynamic parameters using temperature fluorescence data. At 298 K, the Stern–Volmer quenching constant (*K*_*sv*_) was 2.39 × 10^4^ M^−1^, and the Gibbs free energy change (*ΔG*) was −24.98 kJ mol^−1^. The negative *ΔG* demonstrated that the binding of ACN to WPI proceeded spontaneously. The enthalpy change (*ΔH*) and entropy change (*ΔS*) were − 22.00 kJ mol^−1^ and 0.0100 kJ mol^−1^ K^−1^, respectively, indicating that hydrogen bonding and hydrophobic interactions served as the major driving forces for complex formation.

To further verify the above inference at the molecular level and identify specific binding sites, molecular docking simulation was performed as described by Guo et al. (Guo et al., 2025), which is a widely accepted tool for exploring interactions between proteins and bioactive small molecules. β-Lactoglobulin, the predominant component of WPI responsible for ligand binding, was selected as the protein model for docking. The results showed that the optimal binding energies of cyanidin-3-O-glucoside, delphinidin-3-O-rutinoside, and pelargonidin-3-O-glucoside to β-lactoglobulin were − 6.78 kcal/mol, −7.16 kcal/mol, and − 6.77 kcal/mol, respectively, indicating moderate to strong binding affinity. The key amino acid residues involved in binding (Fig. 5A–C) included GLN21, LYS24, ASP112, LYS151, ILE18, THR20, GLU124, and SER126. Specifically, ACN molecules were embedded in the hydrophobic cavity of the protein formed by Trp and Tyr residues, and multiple hydrogen bonds were formed with surrounding polar amino acids, which is highly consistent with the driving forces concluded from thermodynamic analysis. This microscopic evidence corroborates the intrinsic mechanism of fluorescence quenching and further clarifies the interaction mechanism between ACN and WPI at the molecular scale.

### 3.4 UV–vis spectroscopy analysis

UV–Vis spectroscopy can provide insights into interactions among ACN, WPI, and GA. As shown in Fig. 1B, all three nanoparticle formulations exhibited absorption bands in the 200–230 nm range, which were mainly attributed to the π → π* transition of the C=O bonds in the protein peptide backbone (Zhu et al., 2024). Compared with WPI@NP, the maximum absorption peak of WPI-ACN@NP shifted slightly from 278 to 280 nm, suggesting that ACN may promote extension of the WPI peptide chains and induce conformational rearrangements. Such changes would alter the microenvironment of aromatic residues in WPI and produce a red shift, supporting the presence of ACN–WPI interactions. The increased absorbance near 280 nm may also reflect the contribution of ACN itself, which exhibits an absorption maximum at ∼278 nm derived primarily from its aromatic ring structure (Saha et al., 2020). In addition, the absorbance of the binary nanoparticles in the 200–230 nm region was slightly higher than that of WPI@NP, indicating that ACN incorporation enhanced the conjugated system. The conjugated double-bond structures of ACN (e.g., benzene and pyran rings) likely interacted with the WPI backbone, increasing the probability of π → π* transitions. Conversely, the absorbance of GA-WPI-ACN@NP decreased significantly in both the 200–230 and 280–320 nm regions, which may be attributed to GA shielding the aromatic chromophores of both the protein and ACN, thereby limiting ultraviolet exposure (Dong et al., 2024). Specifically, the pronounced decrease in the absorbance of GA-WPI-ACN@NP between 200 and 230 nm may result from cross-linking between GA and WPI that modified protein conformation and from GA coating that masked peptide-backbone C=O groups, reducing the interaction between ultraviolet light and electronic transitions; moreover, GA itself exhibited weak absorption in the short-wavelength region. A similar behavior has been reported for ovalbumin–anthocyanin–gum arabic nanocomposites (Cui et al., 2021). Collectively, these changes indicate binding among ACNs, proteins, and polysaccharides, further confirming the successful ACN encapsulation within the nanocomposite structure.

### 3.5 Fourier transform infrared (FTIR) spectroscopy analysis

**Fig. 2:**
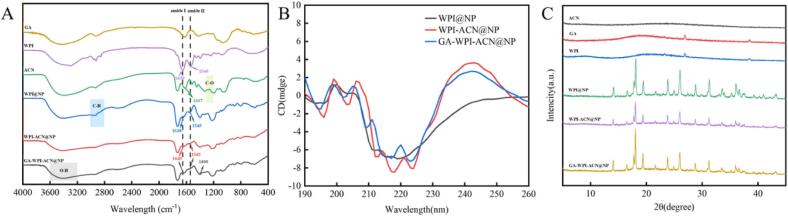
Fourier transform infrared spectra (FTIR, A) Circular dichroism (CD, B) spectra and X-ray diffraction (XRD, C) datterns of individual components and different nanoparticles.

The Amide *I* and Amide II bands are sensitive to changes in protein conformation (DeFlores et al., 2009) As shown in Fig. 2A, the Amide *I* band (C=O stretching) shifted from 1650 to 1637 cm^−1^. In the Amide *I* region, 1648–1664 cm^−1^ is typically assigned to the *α*-helical structure of proteins, while 1615–1637 and 1682–1700 cm^−1^ correspond to *β*-sheet structures. These results indicate that a secondary structural transition occurred during the self-assembly of WPI into nanoparticles, with the conformation changing from *α*-helix and random coil toward *β*-sheet. In addition, the Amide II band (C—N stretching coupled with N—H bending) showed a blue shift from 1540 to 1545 cm^−1^, which confirmed the enhancement of intermolecular interactions. Changes in peak width and intensity in the 2850–3000 cm^−1^ region (C—H stretching) may be related to the enhanced hydrophobic interactions (Zhang et al., 2023). After ACN addition to WPI@NP, the Amide *I* band shifted significantly from 1638 to 1645 cm^−1^, whereas Amide II (C—N stretching vibration and N—H bending vibration) showed almost no change. These spectral shifts were caused by the interactions between WPI@NP and ACN, which perturbed the Amide I band, implying alterations in the *α*-helical structure. Previous studies have (Chanphai et al., 2018) reported that variations in Amide I intensity can arise from polyphenol binding to protein C=O, C—N, and N—H groups (hydrophilic interactions), as well as from changes in protein conformation, consistent with the present findings. After GA incorporation, the characteristic absorption peak of O—H at 3405 cm^−1^ in GA-WPI-ACN@NP showed a significant broadening and enhancement in intensity, confirming successful GA introduction (Wan et al., 2023). Furthermore, the ACN aromatic-ring-associated bands in the 1044–1319 cm^−1^ range (CO stretching) nearly disappeared, further supporting effective ACN encapsulation within the nanocomposite structure. Collectively, the spectral changes demonstrate the successful combination of WPI, ACN, and GA, and these structural features are closely linked to the functional improvements of the nanoparticles detected in this work. The presence of intermolecular interactions and the formation of a composite structure facilitated the encapsulation of ACNs and increased the stability of the final nanoparticles. Based on the structural analysis of other complex lipid and macromolecular systems (Feng et al., 2026), the structural differences among various delivery carriers can be well distinguished, which aids the comprehensive understanding of the structural advantages of the present protein-polysaccharide composite nanoparticles.

### 3.6 Circular dichroism spectroscopy analysis

Interactions between proteins and other molecules can induce changes in protein secondary and tertiary structure. CD spectroscopy is therefore- well suited for investigating protein modifications. Fig. 2B shows the CD spectra of the nanoparticles. WPI@NP exhibited a broad negative band at 205–225 nm, indicating *β*-sheet as the dominant secondary structure. A positive band at 192 nm and two negative bands at 208 and 222 nm correspond to the characteristic peaks of the *α*-helical structure (Wang et al., 2024) Upon ACN incorporation, the reduced intensities at 192 and 208 nm were consistent with the reduction of the *α*-helix content from 12.2% to 7.9%. Concomitantly, the marked enhancement of the negative band at 210–225 nm was accompanied by an increase in the *β*-sheet content from 24.0% to 31.1%, indicating that ACN incorporation altered the secondary structure of WPI@NP and promoted a more stable conformation. Similar observations have been reported previously and have been attributed to ACN–protein structural rearrangements that yield a more stable system (Chen et al., 2019). The negative band around 200 nm is characteristic of random-coil conformations. Notably, GA is a polysaccharide that produces no intrinsic CD signal in the far-ultraviolet region and cannot directly increase the band intensity. After GA coating, the random-coil band exhibited decreased intensity and obvious spectral broadening. This broadening does not originate from GA self-signals but from GA-induced microenvironmental changes and structural rearrangement of surface-exposed protein chains, indirectly confirming the successful GA surface modification. The decreased intensity of this band after GA addition indicated a reduction in the random-coil content. These results suggest that GA interacted with WPI-ACN@NP and altered its random-coil conformational features. A similar effect has also been reported for interactions between oral mucin and GA hydrolysates (Li et al., 2025).

### 3.7 X-ray diffraction (XRD) analysis

XRD is a characterization technique for analyzing the crystalline properties of materials by measuring the interaction between incident X-rays and ordered structures within a material. The XRD patterns of the individual components and the nanoparticles are shown in Fig. 2C. Native WPI is a globular protein with a predominantly amorphous structure, although it can undergo denaturation and aggregation under certain processing conditions (Li et al., 2025). The XRD patterns of WPI, GA, and ACN exhibited diffuse, broad peaks, indicating amorphous crystalline structures (Ye et al., 2021). In contrast, WPI@NP showed multiple sharp diffraction peaks (2θ ≈ 18°, 25°, 31°, and 36°), indicating an increase in crystallinity. This increase may be attributed to the nanonization process, which induced the unfolding and molecular rearrangement and facilitated the formation of ordered secondary structures such as *β*-sheets via self-assembly and hydrophobic interactions (Doan & Ghosh, 2019). The size effects and changes in surface energy may promote local ordering, thereby increasing peak intensity (Doan, et al., 2019). The diffraction peak intensity of WPI-ACN@NP decreased significantly at 2θ ≈ 18° and 25°, and reflections at 2θ ≈ 36° and 38° disappeared, suggesting that ACN loading might interfere with WPI ordering through intermolecular interactions, leading to lattice distortion or partial amorphization (Wan et al., 2023). The diffraction peak intensity of GA-WPI-ACN@NP was further enhanced, particularly at 2θ ≈ 18° and 25°. This enhancement may reflect protein–polysaccharide reorganization after interaction, yielding larger aggregates. Moreover, the rigid structure of GA may restrict molecular motion and stabilize the crystal lattice (Hasanin et al., 2025). Similar increases in crystallinity have been reported for nanoparticles prepared from GA with trypsin and alkaline protease (Mo et al., 2023).

### 3.8 Transmission electron microscopy (TEM)

**Fig. 3:**
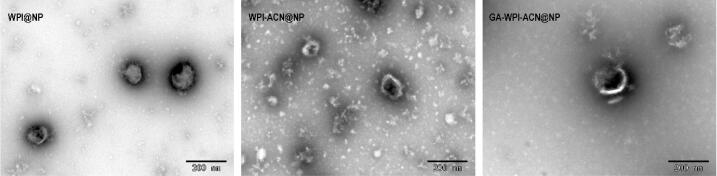
Transmission electron microscopy (TEM) images of different nanoparticles.

In general, the nanoparticle sizes determined by TEM are smaller than those measured by DLS (Khan et al., 2019). This well-documented discrepancy arises because DLS measures the hydrodynamic diameter in an aqueous dispersion, which includes the particle core and the surrounding hydrated layer, whereas TEM visualizes the dehydrated particles after solvent evaporation during sample preparation. As shown in Fig. 3, all three nanoparticle formulations (WPI@NP, WPI-ACN@NP, and GA-WPI-ACN@NP) exhibited spherical morphologies, with sizes smaller than the corresponding DLS values. WPI@NP appeared as regular spherical particles with a smooth, dense surface and uniform particle size. In contrast, WPI-ACN@NP showed a more irregular morphology with a rough surface, likely because ACN loading disrupted the ordered WPI structure and generated heterogeneous microdomains at the particle surface. These microdomains were amorphous complexes formed by binding of ACN polyphenolic moieties to WPI amino and carboxyl groups via hydrogen bonding (Ren et al., 2021). GA-WPI-ACN@NP regained a relatively regular spherical shape and displayed a distinct layered structure, with a dense dark core and a loose annular coating layer on the outside. This indicated that the incorporation of GA filled the microdomains of WPI-ACN@NP and improved the nanoparticle structure. The polysaccharide chains of GA formed a cross-linked network with WPI-ACN@NP through hydrogen bonds, and this network structure could enhance the stability of the nanoparticle core (Cheng et al., 2024). Furthermore, the flexible GA chains extended outward to form a protective layer that inhibited interparticle aggregation (Kan et al., 2024).

### 3.9 Atomic force microscopy (AFM)

**Fig. 4:**
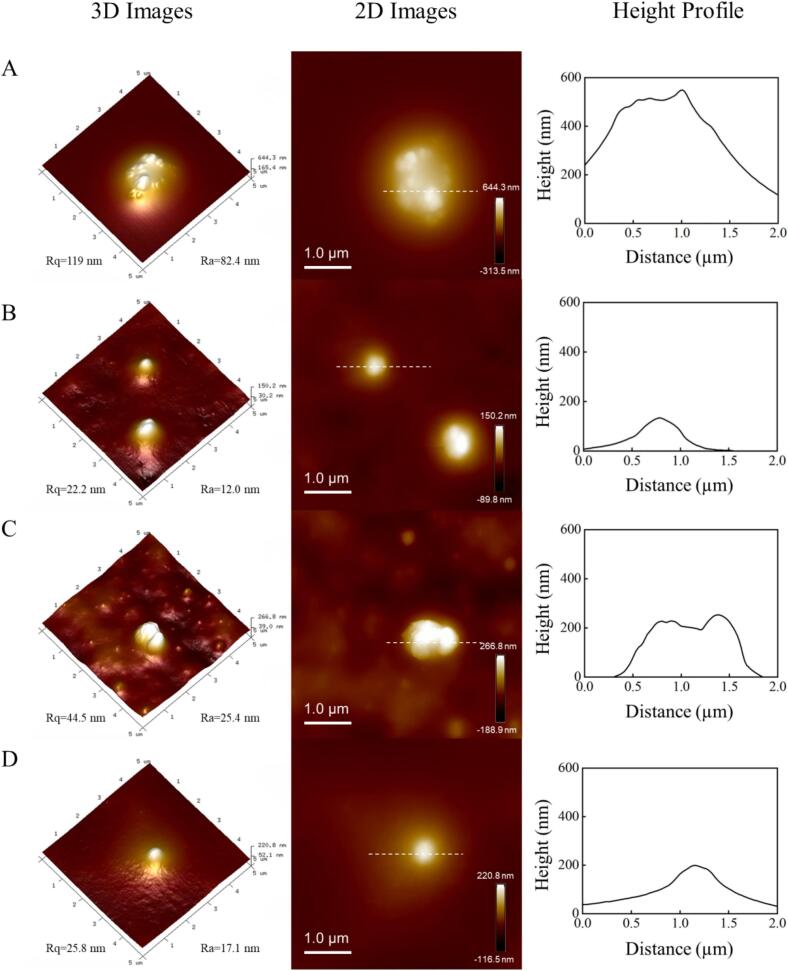
Atomic force microscopy (AFM) images of different nanoparticles (including 2D, 3D and height profile) (A: ACN; B: WPI@NP; C: WPI-ACN@NP; D: GA-WPI-ACN@NP).

Atomic force microscopy (AFM) is a high-resolution tool for morphological characterization that is particularly suitable for investigating the microstructure of individual molecules and complex biomacromolecular composites, providing both two-dimensional (2D) and three-dimensional (3D) morphological information. Fig. 4 shows the AFM images of WPI@NP, WPI-ACN@NP, and GA-WPI-ACN@NP, which exhibited distinct particle sizes and morphologies. Pure ACN showed a surface height of 578.3 nm (Fig. 4A), which is likely attributable to ACN stacking. In contrast, WPI@NP (Fig. 4B) exhibited a relatively low surface height (133.43 nm). Following the formation of binary (WPI-ACN@NP) and ternary (GA-WPI-ACN@NP) nanoparticles, the surface heights were 252.77 nm and 209.1 nm, respectively (Fig. 4C, D). The decrease in height indicated that the nanoparticles could effectively encapsulate ACN and reduce the particle size. Average roughness (Ra) and root-mean-square roughness (Rq) are key parameters for quantifying surface morphology, with Ra reflecting the average deviation degree, whereas Rq is more sensitive to extreme protrusions and depressions (Matsumiya & Murray, 2016). Compared with free ACN, WPI-ACN@NP (Fig. 4C) showed marked reductions in Ra, Rq, and surface height, consistent with ACN incorporation into the nanoparticle matrix and a smoother surface. However, the height profile revealed that the surface of the binary nanoparticles remained heterogeneous, consistent with the TEM observations. Relative to the binary system, the roughness of GA-WPI-ACN@NP was further reduced, demonstrating that GA filled the micro-depressions on the protein surface through its polymer chain structure, formed a more uniform coating layer, and reduced surface undulations (Atgié et al., 2018). These observations confirmed the successful encapsulation of ACNs within the nanocomposite structure.

**Fig. 5:**
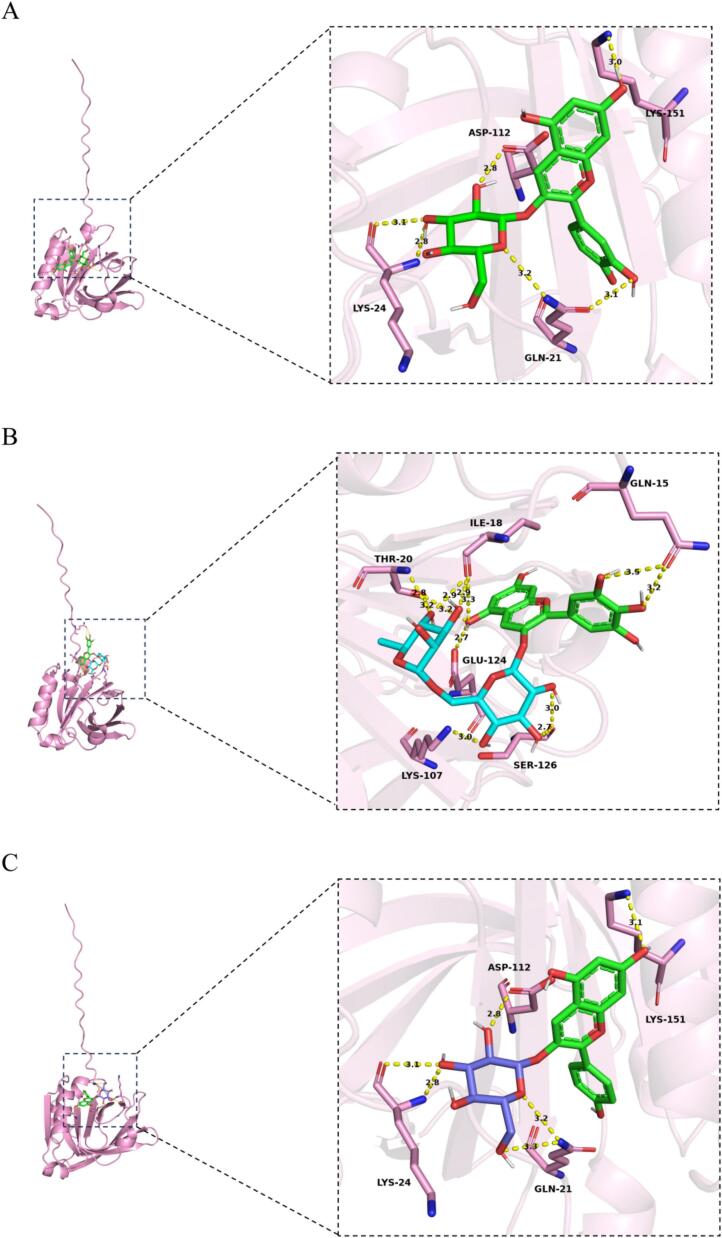
Two-dimensional interaction profiles of *β*-lactoglobulin with three anthocyanin ligands. (A: cyanidin-3-*O*-glucoside; B: delphinidin-3-*O*-rutinoside; C: pelargonidin-3-*O*-glucoside) Each diagram illustrates the types of intermolecular interactions (hydrogen bonds, hydrophobic interactions and van der Waals forces) between the ligand and surrounding amino acid residues, with key binding residues labeled in each subplot.

**Fig. 6:**
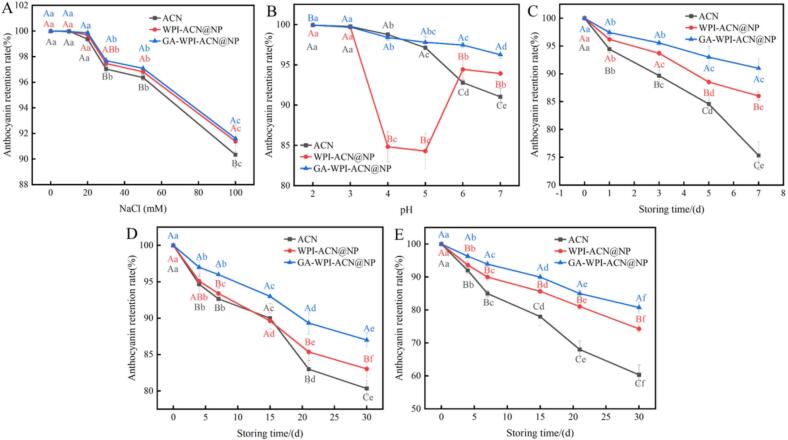
Stability analysis of nanoparticles under different environmental and storage conditions (A: ionic strength stability; B: ph stability; C: light stability; D: 4 °C storage stability; E: 25 °C storage stability). Note: Different lowercase letters indicate significant differences within groups (*p* < 0.05), and different uppercase letters indicate significant differences between groups (p* < 0.05).

**Fig. 7:**
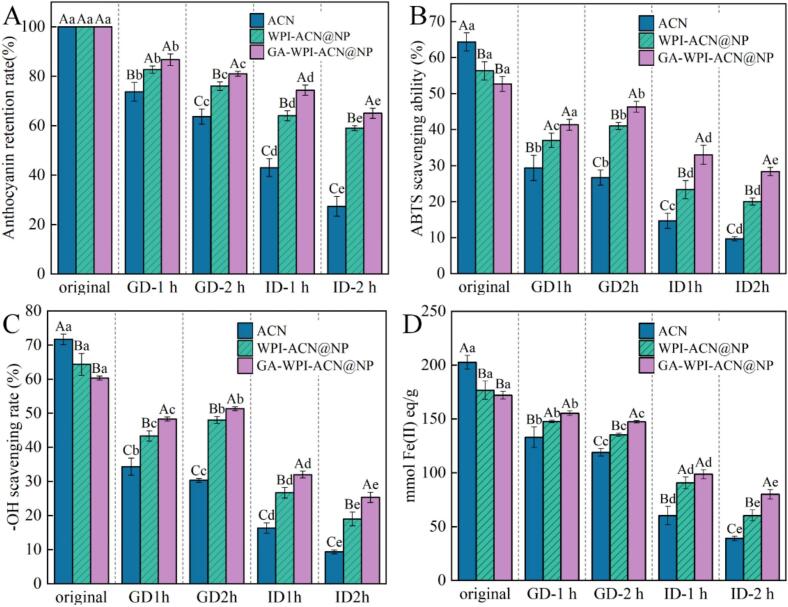
Anthocyanin retention rate and antioxidant activity analysis of different nanoparticles during in vitro simulated gastrointestinal digestion (A: anthocyanin retention rate; B: ABTS free radical scavenging rate; C: hydroxyl radical scavenging rate; D: iron reducing capacity; GD-1 h: gastric digestion for 1 h; GD-2 h: gastric digestion for 2 h; ID-1 h: intestinal digestion for 1 h; ID-2 h: intestinal digestion for 2 h; original: initial state). Note: Different lowercase letters indicate significant differences between groups (p < 0.05), and different uppercase letters indicate significant differences within groups (p* < 0.05).

### 3.10 Environmental and storage stability analysis

Nanoparticle stability under different environmental conditions was investigated as a function of ionic strength, pH, and light exposure. In addition, storage stability at 4 and 25 °C was assessed. As shown in Fig. 6A, increasing NaCl concentration (0–100 mM) decreased ACN retention for all formulations, with GA-WPI-ACN@NP exhibiting the smallest decline. At 100 mM NaCl, ACN retention in GA-WPI-ACN@NP was significantly higher than that of free ACN and uncrosslinked WPI-ACN@NP (*p* < 0.05). Salt ions compressed the electrical double layer on the particle surface through electrostatic shielding, promoting aggregation or structural disruption, particularly for free ACN (Yu et al., 2022). Encapsulation by WPI provided a physical barrier for ACN, while incorporation of GA further enhanced nanoparticle compactness, improving resistance to ionic penetration and charge neutralization. Consistent with prior reports, GA, as an anionic polysaccharide, can stabilize colloidal particles via steric hindrance and thereby reduce salt-induced flocculation (Gao et al., 2024).

As shown in Fig. 6B, all ACN formulations exhibited high stability (retention rate > 95%) under acidic conditions (pH 2–3). However, at pH ≥ 5, free ACN retention decreased significantly (∼90% at pH 7). For WPI-ACN@NP, the primary limitation was the sharp decline in ACN retention at pH 4–5 (*p* < 0.05), which was due to the isoelectric point of WPI (∼4.5–5.2). Under this pH condition, the binary mixture exhibited aggregation and precipitation, resulting in a significant increase in particle size (Fig. S3D, p < 0.05) and a remarkable rise in PDI (Fig. S3E, p < 0.05). This structural instability likely contributed to the reduced ACN retention. In contrast, GA addition mitigated WPI instability near its isoelectric point, and GA-WPI-ACN@NP maintained a retention rate of >95% over the entire pH range. ACN is susceptible to hydrolysis and structural degradation under neutral and alkaline conditions; WPI wrapped ACN via hydrophobic interactions and hydrogen bonding, thereby limiting direct exposure to the external environment (Zang et al., 2021). GA further modulated WPI properties, filled the pores to form a more stable network structure, and inhibited pH-induced conformational changes. Previous studies have shown that WPI–polysaccharide composites can maintain stable zeta potential over a pH range of 1–8 and tolerate high-salt environments (e.g., 200 mM NaCl) (Dai et al., 2015), consistent with the conclusions of this study.

During the 7-day light-exposure period (Fig. 6C), the ACN retention rate of all samples decreased over time; however, GA-WPI-ACN@NP showed the smallest decline and performed significantly better than free ACN and WPI-ACN@NP (*p* < 0.05). By Day 7, the ACN retention rate of GA-WPI-ACN@NP remained above 90%. GA cross-linking increased the mechanical strength of particles by maintaining a stable potential (Fig. S3I), thereby mitigating light-induced structural damage to ACN. Previous studies have shown that WPI–polysaccharide nanoparticles can effectively reduce ACN loss during storage (Chen et al., 2022). In the 30-day storage experiment (Fig. 6D, E), GA-WPI-ACN@NP exhibited the highest stability at both temperatures. Even at 25 °C, the retention rate reached 80.77% ± 1.66% after 30 days, whereas the free ACN showed a retention rate of only 60.33% ± 3.05% (Fig. 6E). Approximately 20% of the ACNs were degraded after 30 days of storage. The partial loss was attributed to the residual endogenous enzymatic activity from the raspberry raw materials and the slow proliferation of trace spoilage microorganisms during storage. This improved retention is indicative of synergistic interactions in the composite carrier: the hydrophobic regions of WPI associate with the hydrophobic groups of ACN, and the GA polysaccharide chains immobilize ACN via hydrogen bonding and covalent cross-linking, thus maintaining a stable potential (Fig. S4C, F). The compact nanoparticle matrix restricts molecular mobility, weakens enzymatic effects, and inhibits microbial growth, thus effectively mitigating ACN degradation (Cheng et al., 2024).

### 3.11 Digestive stability analysis

In vitro simulated digestion (Fig. 7A) showed that GA-WPI-ACN@NP achieved the highest ACN retention during both gastric and intestinal digestion, which was significantly higher than that of the other groups (p < 0.05). The enhanced digestion resistance of the nanoparticles cannot be explained by a single physical barrier effect. Combined with the structural characterization results above, the superior digestive stability originates from the unique hierarchical structure and multiple non-covalent interactions of the composite matrix. As the outermost layer, the GA polysaccharide chains form a dense hydrated film to block gastric acid, intestinal fluid, and digestive enzymes. The intermediate WPI network with rearranged secondary structures possesses good structural rigidity and can resist enzymatic hydrolysis (Tong et al., 2026). ACNs are embedded in the core region and fixed by hydrogen bonds as well as hydrophobic forces. This multiscale structural synergy effectively protects ACNs from degradation in the gastrointestinal environment. The structural advantages of this protein-polysaccharide-ACN composite are similar to those of reported polyphenol-macromolecule complexes, where compact network structures and multi-interactions jointly enhance the tolerance to digestive conditions (Cheng et al., 2024).

### 3.12 Antioxidant activity analysis

As shown in Fig. 7B–D, GA-WPI-ACN@NP showed the maximum retention of antioxidant activity after simulated gastrointestinal digestion (GD/ID treatment). After 2 h of intestinal digestion, the ABTS cation radical scavenging capacity of free ACN decreased sharply to 12.00% ± 0.20% (Fig. 7B). On the other hand, GA-WPI-ACN@NP retained ∼28% of its activity under the same conditions, which was significantly higher than that of WPI-ACN@NP and free ACN (p < 0.05). Similarly, in the hydroxyl radical (·OH) scavenging rate and ferric reducing power assays (Fig. 7C, D), GA-WPI-ACN@NP retained ∼25.00% ± 0.20% activity and 85 mmol Fe (II) eq/g (ferrous sulfate equivalent), respectively, after 2 h of ID treatment, which were significantly higher than those of the other groups (p < 0.05). This improved retention is attributable to crosslinking between the GA polysaccharide chains and the WPI peptide chains via hydrogen bonding and hydrophobic interactions, forming a dense network that effectively encapsulated ACN and enabled sustained release, thereby limiting decomposition under gastrointestinal conditions (Herrera-Balandrano et al., 2021). In the intestinal environment with neutral to alkaline pH, free ACN readily undergoes molecular ring opening and structural damage, leading to loss of bioactivity. Fundamentally, the antioxidant effects of ACN are derived from its characteristic flavonoid structure; the abundant phenolic hydroxyl groups can donate electrons and hydrogen protons to neutralize various reactive oxygen species and nitrogen radicals, while the conjugated double bonds also contribute to radical elimination (Quan et al., 2025; Sadowska-Bartosz & Bartosz, 2024). Only the structurally intact ACN can exert its inherent antioxidant function. Conversely, the GA-WPI carrier significantly slowed ACN degradation by stabilizing the local microenvironment (Kim et al., 2025), resulting in a 2.3-fold increase in ACN retention after 2 h of intestinal digestion. Collectively, these findings indicate that GA-WPI-ACN@NP can efficiently protect ACNs while retaining its molecular structure and antioxidant mechanism during gastrointestinal transit, thus providing a promising strategy for the development of gastrointestinal-stable functional foods.

## 4 Conclusion

In this study, a nanodelivery system encapsulating raspberry ACNs using a WPI–GA composite carrier was successfully constructed. Structural characterization confirmed that the ACNs formed stable core–shell nanoparticles with WPI and GA via hydrophobic interactions, hydrogen bonding, and electrostatic interactions; furthermore, GA incorporation improved the nanoparticle morphology and structural stability. GA-WPI-ACN@NP delivered strong performance in terms of environmental adaptability (broad pH range, high ionic strength, and light exposure conditions), storage stability (long-term storage at 4 and 25 °C), and digestive stability (simulated gastrointestinal environment), significantly mitigating ACN degradation and improving bioaccessibility. In addition, the composite nanoparticles retained strong antioxidant activity after in vitro digestion, confirming that the composite carrier effectively preserved the functional properties of ACNs. After passing through the upper gastrointestinal tract, most ACNs reach the colon and are metabolized by gut microbiota, which is a key link to exert their bioactivities. As demonstrated in previous studies, the composition and structure of macromolecular materials have a remarkable impact on the fermentation characteristics of gut microbiota (Li et al., 2022). The WPI–GA composite carrier can facilitate the gradual release of ACNs, and its protein and polysaccharide components can act as microbial substrates, thus showing potential to modulate intestinal flora. However, all the evaluations in the current study are based on in vitro simulated models, which do not reflect the real physiological environment. In subsequent work, animal models will be used to further validate the in vivo bioavailability and biological effects of this delivery system. Overall, the WPI–GA composite nanocarrier can serve as a feasible tool for the efficient delivery of raspberry ACNs, thus laying a foundation for the development of gastrointestinal-stable functional foods.

## CRediT authorship contribution statement

**Chang Tan:** Writing – review & editing, Visualization, Formal analysis, Conceptualization. **Xue Zhang:** Writing – original draft, Formal analysis, Data curation. **Hanchi Ma:** Writing – review & editing, Investigation. **Chenyang Shi:** Visualization, Resources. **Tiejing Li:** Supervision, Methodology. **Youlin Xue:** Funding acquisition, Conceptualization. **Xue Peng:** Writing – review & editing, Data curation. **Xiaoqian Hu:** Validation, Formal analysis. **Yanwen Kong:** Visualization. **Chongting Guo:** Supervision, Conceptualization. **Mingyue Wang:** Writing – review & editing, Project administration.

## Declaration of competing interest

The authors declare that they have no known competing financial interests or personal relationships that could have appeared to influence the work reported in this paper.

## Data Availability

Data will be made available on request.

## Appendix A Supplementary data

Supplementary material: Supplementary figures and table showing the TIC chromatograms and UPLC-Q-TOF-HRMS identification of components in the purified raspberry anthocyanin extract, together with the particle size distribution and physicochemical stability of the prepared nanoparticles under different ionic strength, pH, light exposure, and storage conditions.
