# Supplementary material for: Construction of an efficient delivery system for raspberry anthocyanins: preparation and stability evaluation of whey protein isolate-gum Arabic nanoparticles
Source: Food Chem X. 2026 Jul 15;38:104216. doi: 10.1016/j.fochx.2026.104216 (PMC13392561; doi:10.1016/j.fochx.2026.104216)
Supplement: Supplementary file 1 — Supplementary material: Supplementary figures and table showing the TIC chromatograms and UPLC-Q-TOF-HRMS identification of components in the purified raspberry anthocyanin extract, together with the particle size distribution and physicochemical stability of the prepared nanoparticles under different ionic strength, pH, light exposure, and storage conditions. [file mmc1.docx]

### **Supplementary Material**

**Supplementary figures and table showing the TIC chromatograms and UPLC-Q-TOF-HRMS identification of components in the purified raspberry anthocyanin extract, together with the particle size distribution and physicochemical stability of the prepared nanoparticles under different ionic strength, pH, light exposure, and storage conditions.**


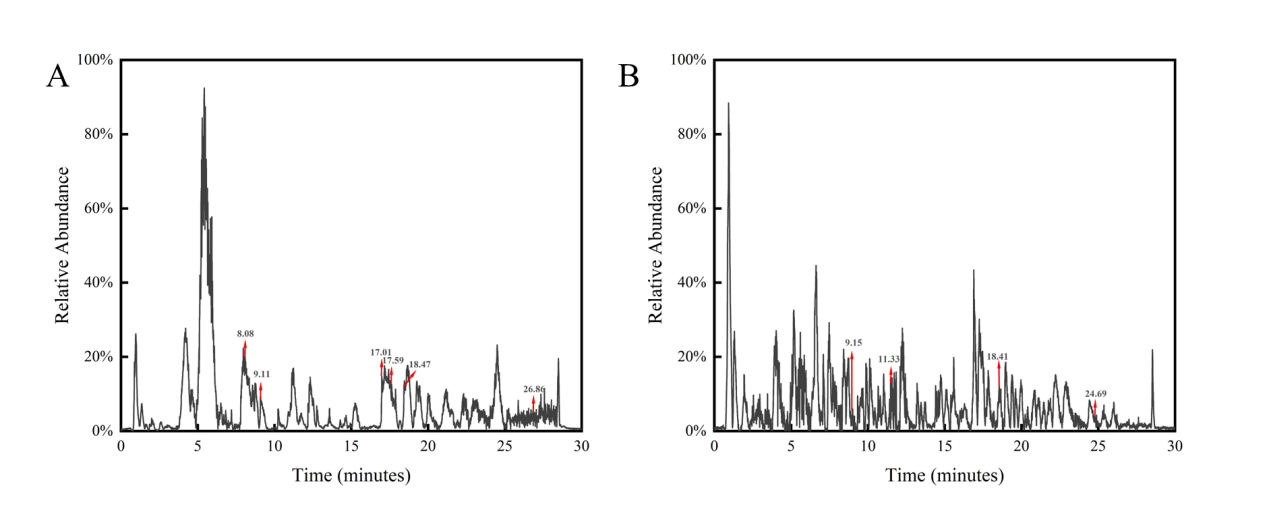


Fig. S1. Total ion chromatograms (TICs) of the purified raspberry anthocyanin extract in (A) positive-ion mode and (B) negative-ion mode.


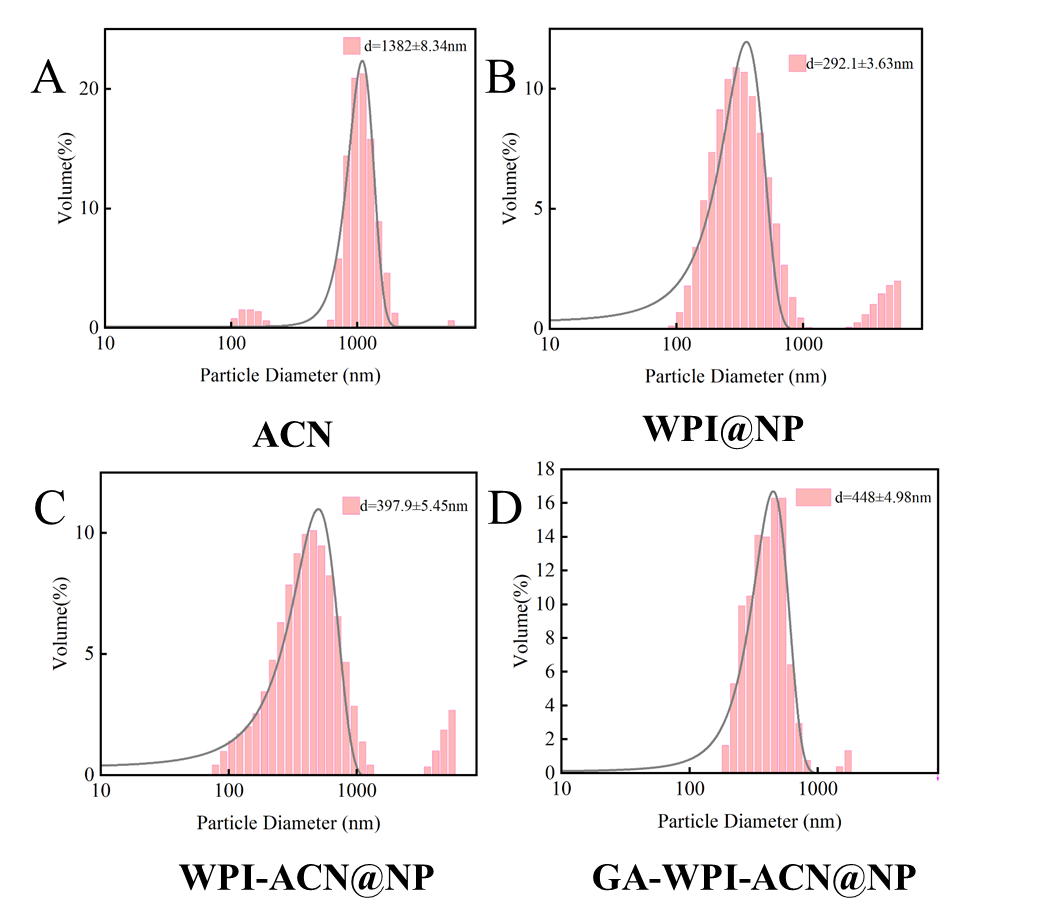


Fig. S2. Particle size distributions of different nanoparticle formulations: (A) ACN, (B) WPI@NP, (C) WPI-ACN@NP, and (D) GA-WPI-ACN@NP.


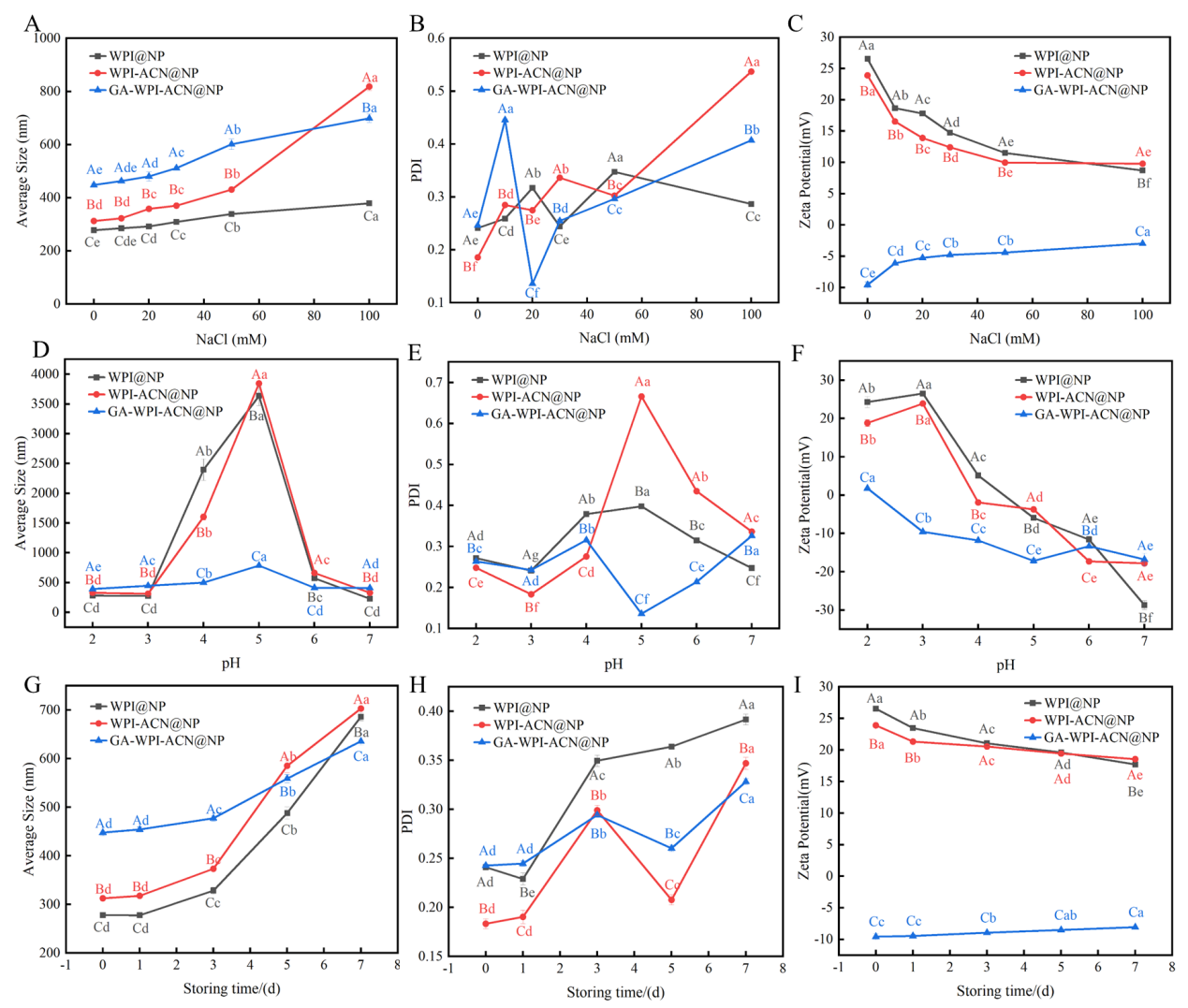


Fig. S3. Changes in the average particle size, polydispersity index (PDI), and zeta potential of different nanoparticles under varying ionic strength, pH, and light-exposure duration: (A–C) ionic strength, (D–F) pH, and (G–I) light-exposure duration; (A, D, and G) average particle size, (B, E, and H) PDI, and (C, F, and I) zeta potential.

Note: Different lowercase letters indicate significant differences among the nanoparticle formulations under the same treatment condition, whereas different uppercase letters indicate significant differences within the same formulation under different treatment conditions (p < 0.05).


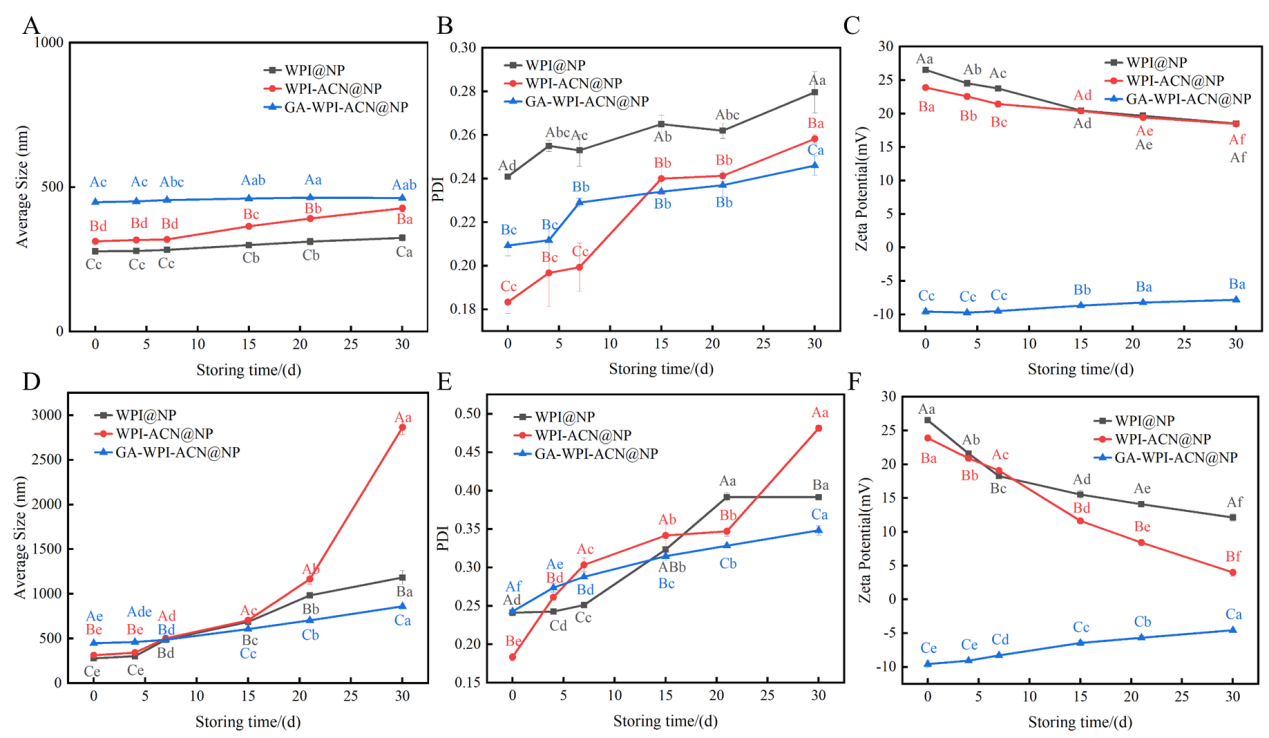


Fig. S4. Changes in the average particle size, polydispersity index (PDI), and zeta potential of different nanoparticles during storage at 4 and 25 °C: (A–C) storage at 4 °C and (D–F) storage at 25 °C; (A and D) average particle size, (B and E) PDI, and (C and F) zeta potential.

Note: Different lowercase letters indicate significant differences among the nanoparticle formulations at the same storage time, whereas different uppercase letters indicate significant differences within the same formulation at different storage times (p < 0.05).

Table S1 Identification results of components in purified raspberry extract via UPLC-Q-TOF high-resolution mass spectrometry

|  | Name | Predicted Molecular Formula | Retention Time | Relative Peak Area (%) | Molecular Ion | ppm | MS/MS | Classification |
| --- | --- | --- | --- | --- | --- | --- | --- | --- |
| 1 | Catechin | C₁₅H₁₄O₆ | 4.218 | 6.92 | 291.0862[M+H]⁺ | 3.76 | 273/151/139 | Flavonoids (Flavan-3-ols) |
| 2 | Epicatechin | C₁₅H₁₄O₆ | 5.874 | 6.53 | 291.0865[M+H]⁺ | 3.91 | 273/151/123 | Flavonoids (Flavan-3-ols) |
| 3 | Procyanidin B1 | C₃₀H₂₆O₁₂ | 8.932 | 5.87 | 579.1588[M+H]⁺ | 4.23 | 425/291/151 | Flavonoids (Proanthocyanidins) |
| 4 | Naringenin | C₁₅H₁₂O₅ | 10.725 | 5.21 | 273.0859[M+H]⁺ | 4.58 | 255/153/107 | Flavonoids (Flavanones) |
| 5 | Hesperetin | C₁₆H₁₄O₆ | 12.387 | 4.98 | 303.0965[M+H]⁺ | 4.12 | 285/165/119 | Flavonoids (Flavanones) |
| 6 | Quercetin-3-*O*-rutinoside | C₂₇H₃₀O₁₆ | 14.821 | 4.65 | 611.1603[M+H]⁺ | 4.79 | 303/179/151 | Flavonoids (Flavonol glycosides) |
| 7 | Genistein | C₁₅H₁₀O₅ | 15.963 | 4.12 | 271.0698[M+H]⁺ | 5.03 | 255/135/118 | Flavonoids (Isoflavones) |
| 8 | Daidzein | C₁₅H₁₀O₄ | 17.215 | 3.87 | 255.0701[M+H]⁺ | 4.87 | 239/135/107 | Flavonoids (Isoflavones) |
| 9 | 1,2,3-Trihydroxybenzene | C₆H₆O₃ | 1.931 | 3.75 | 125.0245[M-H]⁻ | 4.76 | 109/93/79 | Phenylpropanoids |
| 10 | Gallic acid | C₇H₆O₅ | 1.931 | 9.35 | 169.0142[M-H]⁻ | 0.29 | 125/109/79 | Phenylpropanoids |
| 11 | HYDROQUINONE | C₆H₆O₂ | 3.674 | 3.38 | 109.0287[M-H]⁻ | 5.01 | 93/77/65 | Phenylpropanoids |
| 12 | Caffeic Acid | C₉H₈O₄ | 3.98 | 5.72 | 179.0348[M-H]⁻ | 3.98 | 135/117/91 | Phenylpropanoids |
| 13 | Jaboticabin acid | C₁₆H₁₆O₇ | 7.727 | 5.92 | 319.0486[M-H]⁻ | 3.87 | 295/179/135 | Phenylpropanoids |
| 14 | Vanillic acid | C₈H₈O₄ | 7.215 | 4.15 | 167.0293[M-H]⁻ | 4.52 | 123/108/79 | Phenylpropanoids |
| 15 | Ellagic acid | C₁₄H₆O₈ | 8.472 | 5.12 | 301.0007[M-H]⁻ | 5.32 | 275/229/185 | Polyphenols |
| 16 | Spiraeoside | C₂₁H₂₀O₁₀ | 9.853 | 7.12 | 463.0909[M-H]⁻ | 5.13 | 285/159/131 | Flavonoid glycosides |
| 17 | Sinapic acid | C₁₁H₁₂O₅ | 10.574 | 4.89 | 223.0598[M-H]⁻ | 3.99 | 193/178/135 | Phenylpropanoids |
| 18 | Tiliroside | C₃₀H₂₈O₁₂ | 12.344 | 7.85 | 593.1285[M-H]⁻ | 4.21 | 285/159/131 | Flavonoid glycosides |
| 19 | Kaempferol-3-*O*-glucoside | C₂₁H₂₀O₁₁ | 13.257 | 6.58 | 447.0905[M-H]⁻ | 4.98 | 285/159/131 | Flavonoid glycosides |
| 20 | Stigmasterol | C₂₉H₄₈O | 15.103 | 2.78 | 411.3882[M-H]⁻ | 5.03 | 395/377/255 | Terpenoids |
| 21 | 4-Hydroxybenzoic acid | C₇H₆O₃ | 9.628 | 4.45 | 137.0236[M-H]⁻ | 4.19 | 93/77/65 | Phenylpropanoids |
| 22 | LPE 18:1 | C₂₃H₄₄NO₇P | 21.435 | 1.46 | 478.2931[M-H]⁻ | 1.7 | 196/178/281 | Phospholipids |
| 23 | LPE 18:2 | C₂₃H₄₂NO₇P | 20.253 | 0.86 | 476.2782[M-H]⁻ | 0.13 | 196/178/279 | Phospholipids |
| 24 | 2-Isopropylmalic acid | C₇H₁₂O₅ | 4.999 | 0.69 | 175.063[M-H]⁻ | 10.29 | 147/129/85 | Organic acids |
| 25 | FERULATE | C₁₀H₁₀O₄ | 8.36 | 0.06 | 193.0488[M-H]⁻ | 9.5 | 147/132/91 | Phenylpropanoids |
| 26 | Cyanidin-3-*O*-glucoside，tentative | C₂₁H₂₁O₁₁ | 7.356 | 2.85 | 449.1032[M+H]⁺ | 8.56 | 287/179/151 | Flavonoids (Anthocyanins) |
| 27 | Chlorogenic acid isomer，tentative | C₁₆H₁₈O₉ | 9.874 | 1.45 | 355.1036[M+H]⁺ | 12.84 | 191/173/135 | Phenylpropanoids (Chlorogenic acid derivatives) |
| 28 | Delphinidin-3-*O*-rutinoside，tentative | C₂₇H₃₁O₁₇ | 11.628 | 2.37 | 611.1605[M+H]⁺ | 9.32 | 303/179/153 | Flavonoids (Anthocyanins) |
| 29 | Tentatively identified：Quercetin-3-*O*-galactoside，tentative | C₂₁H₂₀O₁₁ | 13.582 | 1.98 | 465.0958[M+H]⁺ | 10.15 | 303/179/151 | Flavonoids (Flavonol glycosides) |
| 30 | Tentatively identified：Kaempferol-3-*O*-rutinoside，tentative | C₂₇H₃₀O₁₅ | 15.721 | 1.62 | 595.1502[M+H]⁺ | 11.28 | 285/159/131 | Flavonoids (Flavonol glycosides) |
| 31 | Tentatively identified：Quercetin-3-*O*-glucuronide，tentative | C₂₁H₁₈O₁₃ | 6.925 | 1.87 | 477.0638[M-H]⁻ | 11.85 | 303/179/151 | Flavonoids (Flavonol glycosides) |
| 32 | Tentatively identified：Caffeoylquinic acid isomer，tentative | C₁₆H₁₈O₉ | 7.853 | 1.52 | 353.0872[M-H]⁻ | 13.26 | 191/173/135 | Phenylpropanoids |
| 33 | Tentatively identified：Luteolin-7-*O*-glucoside，tentative | C₂₁H₂₀O₁₁ | 10.368 | 1.29 | 447.0902[M-H]⁻ | 14.57 | 285/159/135 | Flavonoid glycosides |
| 34 | Tentatively identified：Pelargonidin-3-O-glucoside，tentative | C₂₁H₂₁O₁₀ | 15.621 | 1.15 | 431.0875[M-H]⁻ | 12.93 | 271/155/137 | Anthocyanins |

Note: Only core anthocyanin compounds with a relative peak area ≥ 1.5% are presented in the table. Compounds 1–28 were unambiguously identified with mass errors below 5 ppm. Due to relatively high mass deviations (>10 ppm) and insufficient MS/MS fragment evidence, compounds 29–34 were classified as tentatively identified components.
